# Porous fiber materials can alleviate the risk of farmland drought and flooding disasters and prompt crop growth

**DOI:** 10.3389/fpls.2023.1201879

**Published:** 2023-10-12

**Authors:** Tianling Qin, Shanshan Liu, Wei Li, Shu Xu, Jie Lu, Zhenyu Lv, Sintayehu A. Abebe

**Affiliations:** ^1^ State Key Laboratory of Simulation and Regulation of Water Cycle in River Basin, China Institute of Water Resources and Hydropower Research, Beijing, China; ^2^ Hydraulic and Water Resources Engineering Department, Debre Markos University Institute of Technology, Debre Markos, Ethiopia

**Keywords:** rock wool, runoff, soil water content, nitrogen and phosphorus loss, microorganism, crop growth

## Abstract

Floods and droughts on farmland seriously damage agricultural production. Porous fiber materials (PFM) made from mineral rocks have high porosity, permeability, and water retention and are utilized widely in green roofs and agricultural production. Therefore, studying the impact of PFM on the improvement of farmland is of great importance for soil and water conservation. We set 64 extreme rainfalls to analyze the impact of PFM on soil water content (SWC), runoff, nutrient loss, microorganism, and plant growth. The results showed that PFM can effectively reduce runoff and improve soil water distribution, and enhance the soil water holding capacity. Furthermore, PFM reduced the loss of nitrogen and phosphorus by 18.3% to 97% in the runoff, and the soil erosion of summer corn was more strongly influenced by lower vegetation cover, compared with winter wheat. Finally, when PFM was buried in the soil, the wheat yield increased by −6.7%–20.4%, but the corn yield in some PFM groups decreased by 5.1% to 42.5% under short-duration irrigation conditions. Our study emphasizes that the effectiveness of PFM depends mainly on the following: First, PFM with high porosity can increase soil water holding capacity and timely replenish the water lost from the surrounding soil. Second, PFM with high permeability can increase infiltration during rainfall and decrease runoff and nutrient loss, reducing the risk of farmland flooding and pollution. Finally, PFM consists of gold ions and alkali metal oxides, which can stabilize agglomerates and improve soil enzyme activity, thereby increasing the relative abundance of some microbial strains and promoting crop growth. However, when the rainfall amount was low or PFM volume was large, PFM could not store water sufficiently during rainfall, which seriously reduced the maximum saturated moisture content and water absorption performance. Meanwhile, the PFM could not release water in time and replenish the soil water deficit, which increased drought risk. In conclusion, the appropriate volume of PFM and irrigation system may enhance soil water storage capacity, minimize agricultural pollution, and promote crop production.

## 1 Introduction

To meet the food demand of the increasing population, people change land use and over-exploit arable land, which has reduced the soil water storage capacity of farmland, and the massive use of fertilizers and pesticides has increased farmland pollution, both of which have seriously damaged farmland ecosystems (Romero et al., 2020; Winkler et al., 2021). In addition, food production faces enormous competitive risks from land, water, and energy (Gathala et al., 2020). It is crucial to study how to effectively use the soil, water, and plant resources in farmland under different cropping conditions, maintain the stability of farmland ecosystems, and promote food production (Liu et al., 2020b).

Many water-absorbing materials are currently used as soil conditioners in slopes, farmland, and green roof, and they have been proven to be one of the most effective strategies for promoting the stability of farmland ecosystems (Sadeghi et al., 2020; Wang et al., 2022a). For example, biochar and straw can reduce soil bulk and increase soil pore, allowing the soil to maintain a high hydraulic conductivity and water storage capacity, thereby alleviating drought and flooding on farmland (Zhang et al., 2021b; Adhikari et al., 2022). In addition, biochar, with abundant oxygen-containing functional groups, can absorb some elements and heavy metal ions through chemical bonds and other forces, thus reducing the risk of environmental pollution in agricultural soils (Shaaban et al., 2018). Nevertheless, the above traditional water-absorbing materials only act on the tillage layer, lessening the effect on the deep soil and not meeting the regulation needs (Lv et al., 2020). For example, scholars have found that the application of biochar in coarse soil may block soil pores or be easily washed away by surface runoff, which seriously increases the risk of soil erosion and ecological damage to farmland (Li et al., 2019; Li et al., 2020; Murtaza et al., 2021).

Porous fiber materials (PFMs, www.hydrorock.com) are composed of natural mineral rocks with properties similar to biochar, including high porosity, water retention capacity, and stability. PFM is made in the following way: the raw material is melted at 1600°C, centrifuged at high speed, sprayed with a special reagent, and finally fixed and cut (Lv et al., 2020). The average price of PFM is 1000 CNY/m^3^–2000 CNY/m^3^. Since rock wool was developed by the Grodan Company in Denmark in 1969, it helped precisely control the water, air, and fertilizer ratio for plant roots in drip irrigation systems. The buffering effect on external environmental humidity changes can create a stable growth environment for plant root growth. As a result, it has been widely used as a substrate for seedling and nutrient cultivation in soilless cultures (Xiong et al., 2017; Choi and Shin, 2019). In detail, Bussell thought that PFM could accommodate more water than other media, owing to its porosity of over 90% (Bussell and McKennie, 2004).

As PFM gradually transitions from a saturated to an unsaturated state, substrate water tends to move more horizontally, which helps PFM regulate the farmland’s soil environment (Silva et al., 1995). Lv had studied that PFM acts as a water storage module in farmland, could improve soil structure, increase infiltration, and reduce runoff, ultimately scavenging polluting elements and promoting soil and water conservation (Lv et al., 2020; Lv et al., 2021; Li et al., 2023). At the same time, as an inorganic mineral material with rich pores and a large specific surface area, PFM contains various particles, ions, humus, and silicates, which facilitate the formation of soil agglomerates and nutrient supply (Kováčik et al., 2010). In addition, Gu showed that PFM could effectively intercept natural rainfall and release water slowly to prompt the growth of forestland plant, which could mitigate the inhibition of plant morphological growth by water stress (Gu, 2021), and increases tomato plant height, ground stems, leaf area, root length, and biomass in the pot trials too (Gu, 2021). Therefore, the development and application of PFM in water retention technology are essential to improve soil water retention capacity in the field, optimize plant water management, and prompt crop growth, which is important to further expand the application fields of PFM such as agricultural production, flower cultivation, and urban greening (Li et al., 2022).

**Figure 1:**
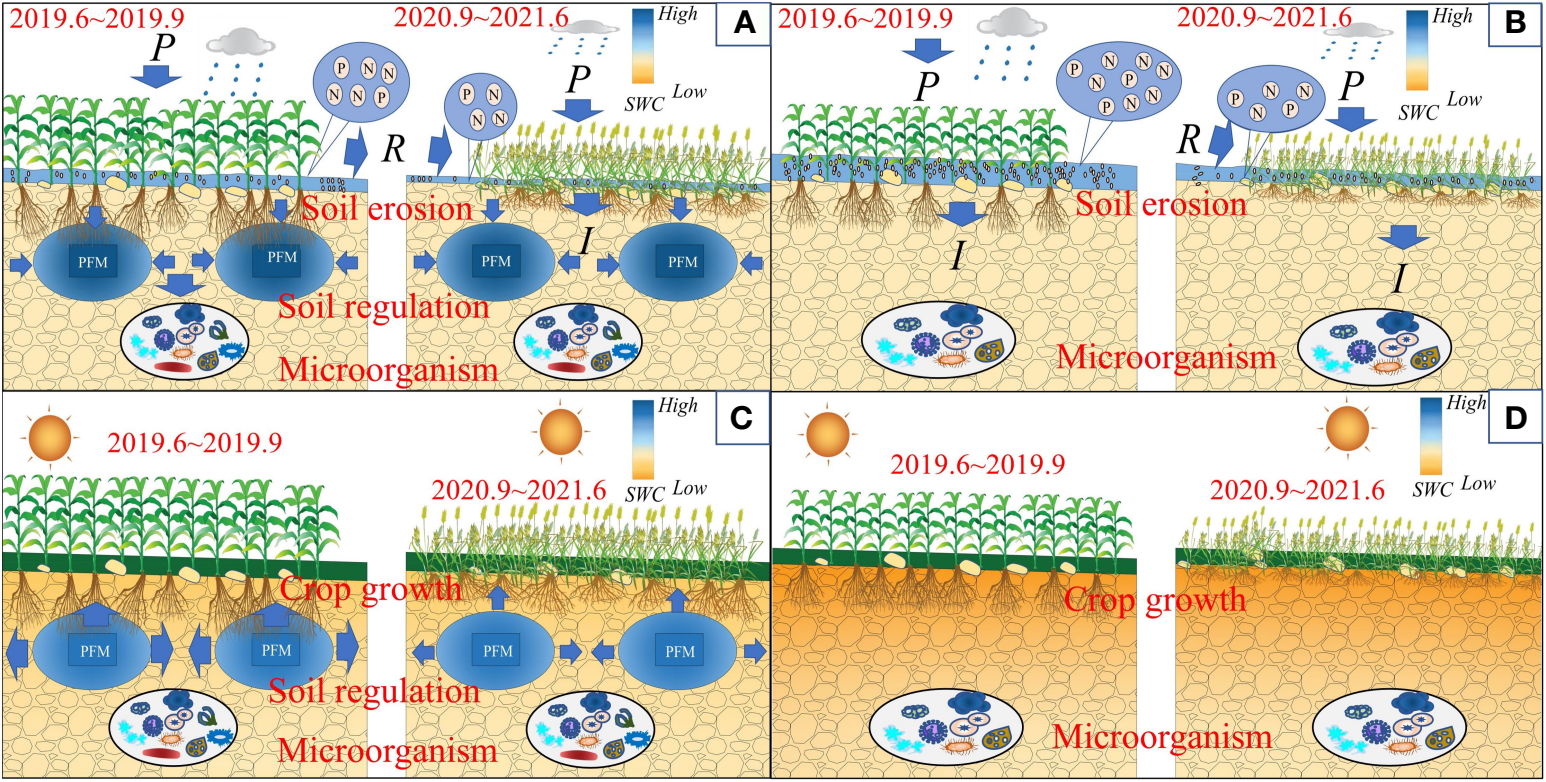
Scientific hypothesis. PFM embedding in the farmland increases soil porosity, which benefits soil water content (SWC) and nutrient distribution, ultimately promoting crop production. **(A–D)** represent the variation of SWC, crop growth, microorganisms, and nutrient loss, respectively.

Nevertheless, different crops have different characteristics in the root physiological and biochemical aspects, which make the application of soil conditioners show significant differences in effectiveness (Hou et al., 2021; Laub et al., 2022). Meanwhile, due to the limitations of research conditions and technology, most field studies only have focused on a single aspect of the effects of PFM, including infiltration, soil water variability, crop growth, and material properties (Lv et al., 2020; Gu, 2021; Lv et al., 2021). However, there are few studies on the comprehensive effects of PFM on farmland ecology under different crop cultivation conditions. So, there is an urgent need for deep exploration. Therefore, we hypothesize that rock wool materials can regulate and meet crops’ water needs under external water stress. Their excellent pore structure and water retention can reduce nitrogen and phosphorus loss and promote soil nutrient retention, finally improving the farmland soil environment and increasing crop production (**Figure 1**).

As an important grain-producing area in Anhui Province, the principal crops cultivated in the Huaibei Plain are wheat, corn, soybeans, sorghum, and so forth. The crop production of Huaibei Plain in 2021 is 2.25 × 10^7^ tons, accounting for about 55% of the total crop production in Anhui Province. However, due to the uneven spatial and temporal distribution of rainfall and other factors, more than 1.73 million hectares, or 83.1% of Huaibei Plain’s arable land, are located in the flood-prone area. In addition, flooding frequently occurs with soil nitrogen, phosphorus, and heavy metal elements migrating with surface runoff and leaching, leading to serious eutrophication of rivers and lakes and groundwater pollution. The shallow groundwater level exceeded the recommended value of NO_3_-N by the Chinese drinking water standard in 2005 (Qian et al., 2015; Liu et al., 2017; Wei et al., 2023). Overall, it is crucial to research ways to improve soil water storage capacity to lower the risk of droughts and floods, reduce agricultural surface pollution, and promote crop growth. The objectives of the present study were to (1) discover the effects of PFM on farmland runoff, soil water content (SWC), nitrogen, and phosphorus losses, microorganisms, and crop growth and (2) explore the ability of PFM to cope with water stress under different cropping conditions. The above studies can provide practical support to mitigate drought and flood risks and improve crop yields.

## 2 Study area and method

### 2.1 Study area

**Figure 2:**
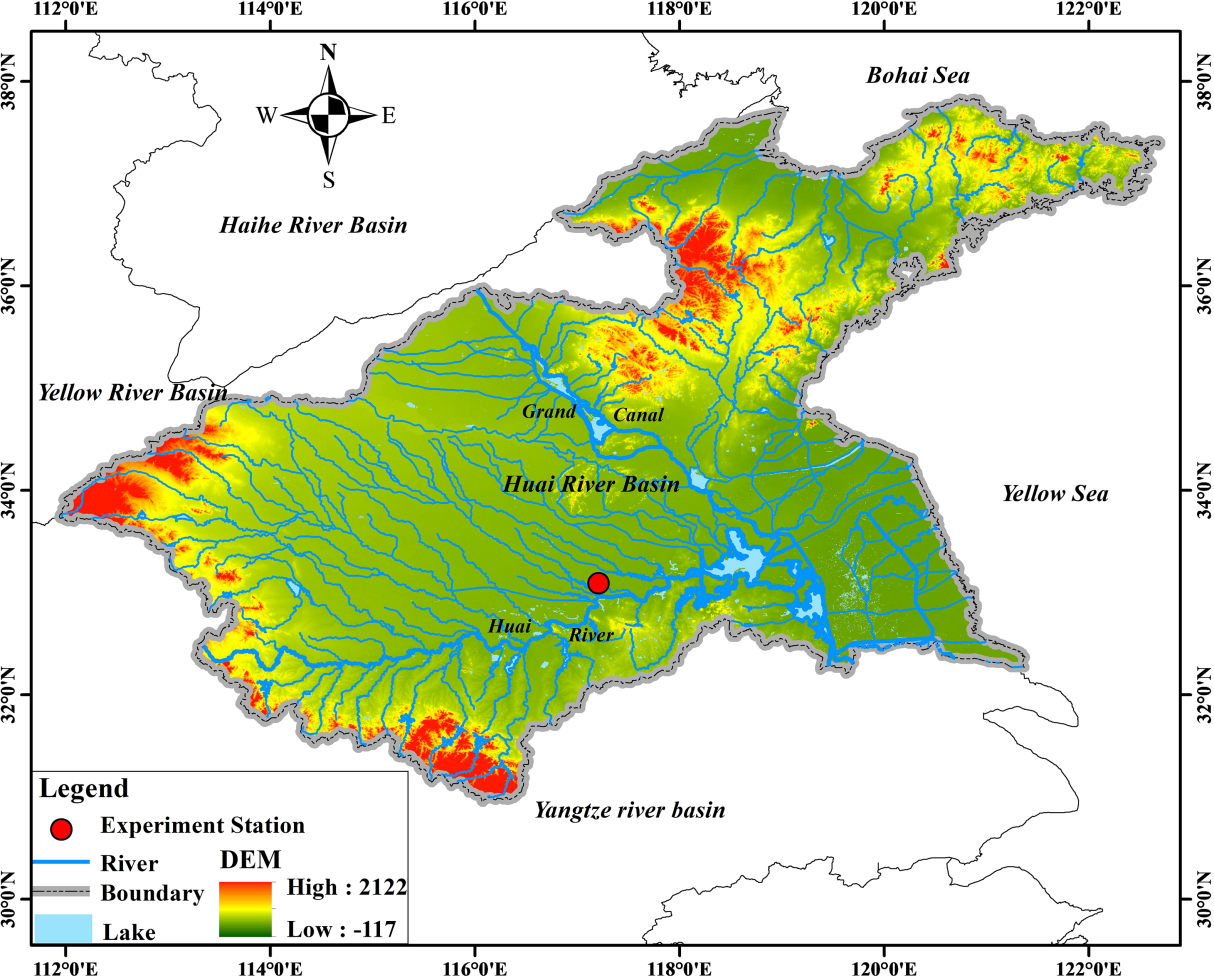
Location of the experiment station.

The experiments were conducted at Wudaogou Hydrological Station (117°21′E and 33°09′N) in Bengbu City, Anhui Province, China. The region is located in the Huai River basin (**Figure 2**), which has a north subtropical and warm temperate semi-humid monsoon climate, with an average annual temperature of 14.7°C, an average annual precipitation of 890 mm, and an annual average rainfall–runoff of 240.2 mm. The maximum rainfall intensity in history was approximately 92.4 mm/h (Bi et al., 2019; Gou et al., 2020).

The soil in the experimental station is a lime concretion black soil with a sticky texture and insufficient organic matter content from 5 g/kg to 15 g/kg. The soil’s effective depth in this region is around 100 cm, and its porosity is about 49.7%. The average soil bulk density is 1.5 g/cm^3^, and the average contents of sand, silt, and clay are 29.6%, 37%, and 33.4%, respectively. The average field capacity and wilting point are approximately 34%Vol and 15%Vol, respectively (Lv et al., 2020). Due to the uneven spatial and temporal distribution of water supplies and poor soil structure, the Huaibei Plain, a significant grain-producing region, is vulnerable to droughts and floods, which can easily reduce crop yields (Liu et al., 2017).

### 2.2 Porous fiber material

PFM is made of natural mineral rocks. PFM is made in the following way: the raw material is melted at 1600°C, centrifuged at high speed, sprayed with a special reagent, and finally fixed and cut. The main components of PFM include metal and non-metal oxides such as Fe_2_O_3_, Al2O_3_, MgO, CaO, and SiO_2_. In detail, PFM has a cross-fiber arrangement, a porosity range of 94%–96%, a compressive strength between 5000 g/cm^2^ and 7400 g/cm^2^, and a permeability coefficient of 5 to 8.5 mm/s.

When the rain stops, SWC progressively drops to a non-saturated state via infiltrating, evaporating, or being absorbed by the plant roots. Because the soil has a significantly larger capillary force than PFM, it will continue to absorb water from the PFM until it is empty. It is conducive to the in situ dissipation of rainwater and surface water. Due to the extremely low capillary, PFM will not absorb water from the soil when the SWC is low or dry, eliminating the possibility of aggravating soil drought or water shortage.

### 2.3 Experiment design

#### 2.3.1 Variables

To enhance soil water holding capacity, we explored the applicability of the PFM on farmland by artificial rainfall experiment. We set four experimental variables based on local environmental conditions: PFM volume, rain intensity, crop growth period, and crop type. Eight plots and 64 rainfall experiments were set based on the above factors listed in **Table 1**.

**Table 1:** Experiment design.

Experiment group	PFM volume (m^3^)	Rainfall intensity (mm/h)	Rainfall (mm)	Duration (h)
A1	0	100	100 (150)	1 (1.5)
A2	1.08	100	100 (150)	1 (1.5)
A3	2.16	100	100 (150)	1 (1.5)
A4	3.24	100	100 (150)	1 (1.5)
B1	0	50	100 (150)	2 (3)
B2	1.08	50	100 (150)	2 (3)
B3	2.16	50	100 (150)	2 (3)
B4	3.24	50	100 (150)	2 (3)

Numbers in the bracket represent the rainfall amount and duration in the winter wheat experiment, others represent the summer maize experiment. The detail refers the section 2.3.1.2, please.

##### 2.3.1.1 PFM volume

**Figure 3:**
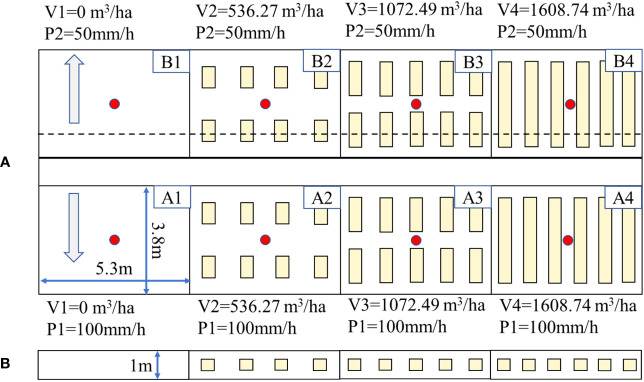
Location of the porous fiber material (PFM) in experimental plots. Pictures **(A, B)** represent the plane and sectional figures of PFM embedding, respectively. A1, A2, A3, and A4 groups represent the experimental groups under 100 mm/h rainfall events. B1, B2, B3, and B4 groups represent the experimental groups under 50 mm/h rainfall event; the arrow represents the direction of the slope.


In agricultural production, soil pore is closely related to infiltration and water movement, and soil water holding capacity is generally related to porosity in a power function. Therefore, this study was set to increase the soil water-holding capacity of the experimental plot by 0%, 5%, 10%, and 15%, referring to the “Sponge City Construction Technical Guide” of China. According to the formula, the volume of PFM embedding was set to 0 m^3^/ha, 536.27 m^3^/ha, 1072.49 m^3^/ha, and 1608.74 m^3^/ha. Correspondingly, the area of a single experimental plot was 20.14 m^2^, so the volume of PFM embedding was 0 m^3^, 1.08 m^3^, 2.16 m^3^, and 3.24 m^3^ in the A1–A4 and B1–B4 groups, respectively. The PFM has three specifications: 0.75 m^3^ × 0.45 m^3^ × 0.4 m^3^ (length, width, and height, A2 and B2 groups), 1.0 m^3^ × 0.45 m^3^ × 0.4 m^3^ (A3 and B3 groups), and 1.2 m^3^ × 0.45 m^3^ × 0.4 m^3^ (A4 and B4 groups, **Figure 3**). The calculation formula is shown below (Lv et al., 2020):

(1) $$\delta =\frac{V_{1}}{V_{0}}\times \frac{\beta_{1}-\beta_{0}}{\beta_{0}}\times 100\%$$

(2) $$V_{0}=L\times D\times H$$

Where δ represents the theoretical increase in the soil water-holding capacity of the experiment plot, %; *V*_0_ and *V*_1_ represent the volume of the experimental plot and PFM, respectively, m^3^; β_0_ and β_1_ represent the porosities of the soil and the PFM, respectively, %; *L*, *D*, and *H* represent the length, width, and effective depth of experimental plot, m.

##### 2.3.1.2 Rainfall intensity

The rainfall intensity was set at 100 mm/h and 50 mm/h (**Table 1**), according to the rainfall event records of the typical dry, normal, and wet years of the experimental station. Their return periods were 100 and 30 years in the Huaibei plain, and all represented a grade of heavy rain (the historical maximum rainfall intensity was 92 mm/h). Referring to the pre-experimental results, to ensure that rainfall experiments have similar runoff durations in different crop conditions, the rainfall amount of the summer maize experiment was set to 100 mm, and the total rainfall durations with the 100 mm/h and 50 mm/h rainfall intensities were 1h (high rainfall intensity and short duration events) and 2h (low rainfall intensity and long duration events), respectively. The rainfall amount of the winter wheat experiment was set to 150 mm, and the total rainfall durations with the 100 mm/h and 50 mm/h rainfall intensities were 1.5h (high rainfall intensity and short duration events) and 3h (low rainfall intensity and long duration events), respectively.

##### 2.3.1.3 Growth period

**Figure 4:**
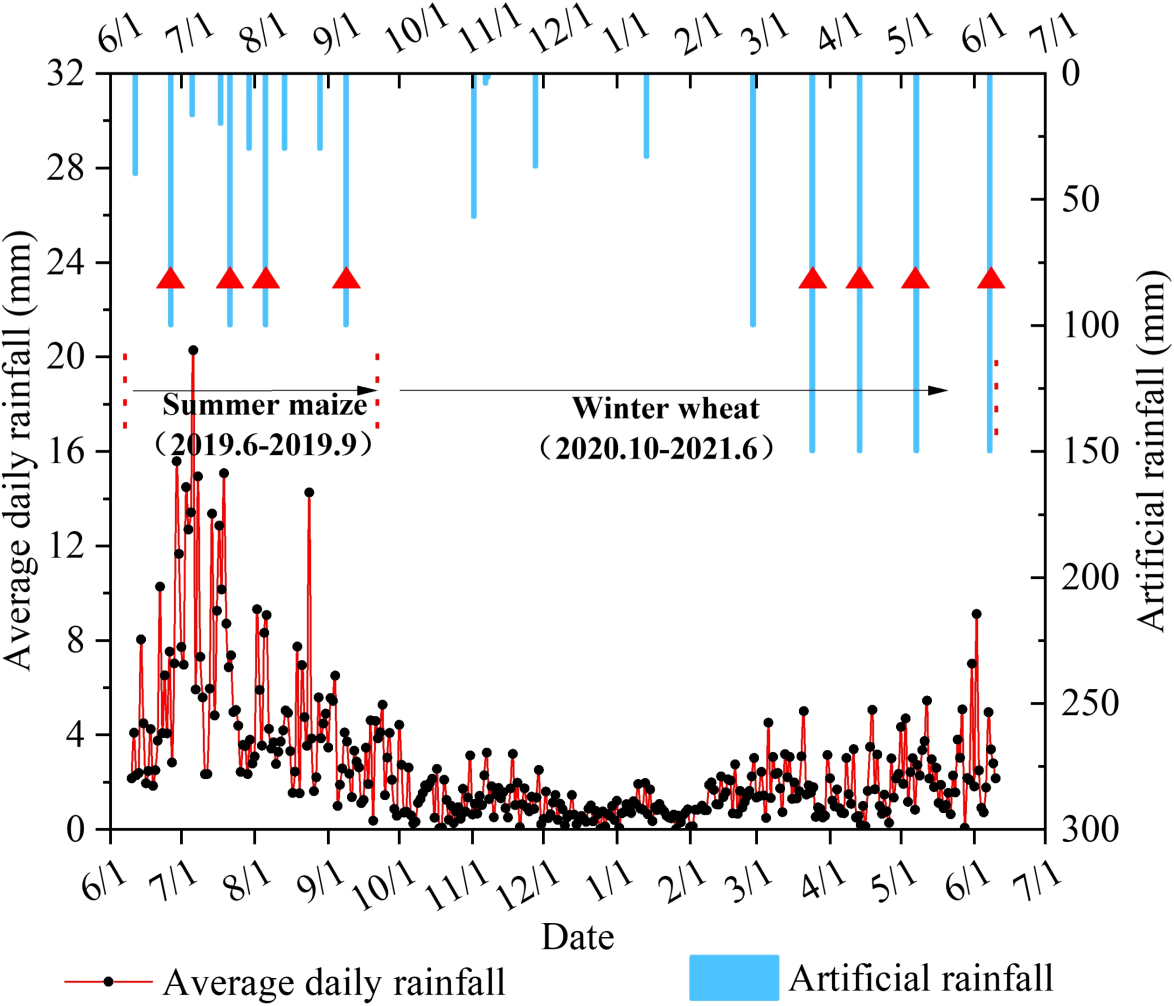
Rainfall data (black dots represent the multi-year average daily rainfall. The rainfall events with a triangle are rainfall runoff experiments, and the rest are rainfall recharge events).

Referring to the water demand intensity of crops and the natural rainfall period, we set some artificial rainfall events during the growth period, and the rainfall events included the irrigation recharge and rainfall runoff experiments. In contrast, the irrigation recharges were aimed at ensuring crop growth normally, but the rainfall–runoff experiments were for exploring the application of the PFM to farmland flooding. According to the water requirement of winter wheat in the Huaibei Plain recorded by the experimental station, irrigation recharge was carried out according to the average rainfall for many years before the greening period. The aim was to ensure the normal growth of winter wheat, so we irrigated at seedling, tillering, overwintering, and re-greening stages with 57 mm, 37 mm, 33.2 mm, and 100 mm of irrigation water, respectively. The rainfall–runoff experiment was conducted at the heading stage (Day 144), flowering stage (Day 165), grouting stage (Day 189), and fallowing stage (Day 220) with 150 mm of rainfall. Similarly, the rainfall–runoff experiment for the summer maize was conducted at the emergence (Day 15), nodulation (Day 40), tasseling (Day 54), and maturity stages (Day 89), with 100 mm of rainfall, respectively. Additionally, irrigation recharges were carried out on days 1, 33, 62, and 77 with 40 mm, 16.6 mm, 30 mm, and 30 mm of irrigation water, respectively (**Figure 4**).

#### 2.3.2 Experimental plot

**Figure 5:**
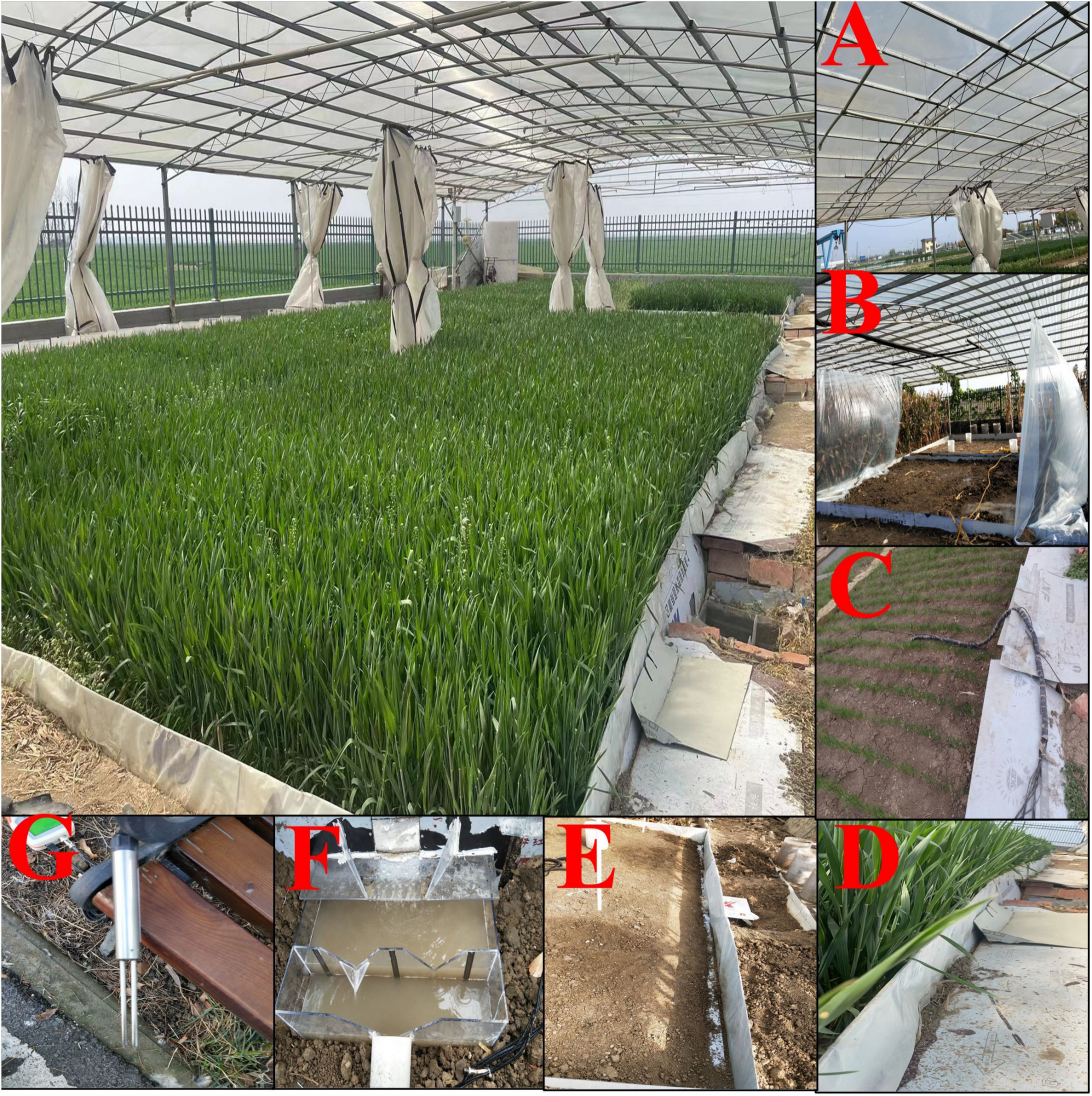
The experimental plot and its composition. Figures **(A–G)** represent the composition of the whole experimental group. It includes artificial rainfall devices **(A)**, wind dodge **(B)**, rain-stop cover **(C)**, water-stop sheet **(D)**, runoff channel **(E)**, water tank **(F)**, and moisture sensor **(G)**.

The experimental plots are designed considering the local topography, and each experimental plot is set with a 3° gradient and a size of 5.3 m^2^ × 3.8 m^2^ in the north–south direction (**Figure 3**). Every experimental plot is composed of an artificial rainfall device (**Figure 5A**), wind dodger (**Figure 5B**), rain-stop cover (**Figure 5C**), water-stop sheet (**Figure 5D**), runoff channel (**Figure 5E**), water tank (**Figure 5F**), and water moisture sensor (**Figure 5G**). Rainfall devices are installed at the height of 4 m from the land surface, and they include four conical nozzles and produce a controlled rainfall intensity ranging from 30 mm/h to 150 mm/h. The wind dodger hung around the experimental plots is a transparent, waterproof, and polyethylene cloth; the water-stop sheet is set to 100 cm according to the local soil effective depth, and the depth of PFM embedding is set to 30 cm–70 cm; the runoff channel is located at the bottom of the slope of every experimental plot with dimensions of 5.3 m^3^ × 0.15 m^3^ × 0.1 m^3^, and bottom of the channel is buried with 20 cm deep sand. The water tank was made of organic glass, with a size of 0.6 m × 0.45 m × 0.3 m (width, length, and height), and the runoff amount was recorded by an isosceles-triangular weir. Meanwhile, soil water sensors are buried at 0.2 m, 0.4 m, and 0.6 m depths at the center of the experimental plot, respectively, which aim to measure the SWC change during the experiment (Lv et al., 2020; Li et al., 2022). For more details, please refer to previous papers by (Lv et al., 2020; Li et al., 2022).

#### 2.3.3 Field management

Referring to the local planting system, during the summer maize (name: Deng-Hai 618) experiment (2019.6–2019.9), we applied the diammonium (18% N and 46% P), urea (46% N), and red potash (50%–60% K) at 300 kg/ha, 400 kg/ha, and 250 kg/ha before planting, respectively. Then, we planted summer corn in June 2019, and the planting density of summer corn was set at 6.7 plants/m^2^, while fertilizer was chased at 150 kg/ha of urea between the jointing period and the tasseling period. Furthermore, during the winter wheat (name: Huai-Mai 45) experiment (2020.11–2021.6), we applied the compound fertilizer (24% N, 15% P, and 6% K) and urea (46% N) at 750 kg/ha and 150 kg/ha before planting, respectively. Then, we planted winter wheat on 1 November 2021, and fertilizer was chased at 75 kg/ha of urea during re-greening. The remaining field management, such as insecticide, weed control, and micro fertilizer spraying, was the same as the field.

### 2.4 Data measuring

#### 2.4.1 Soil water content

During the experiment, we monitored the SWC at 8:00 a.m. and 8:00 p.m. every day by soil sensors, and the SWC was determined with a portable FDR probe at 20 cm, 40 cm, and 60 cm soil depth, respectively.

#### 2.4.2 Runoff process

We pre-rained before planting, which fully saturated the SWC and prompted seed germination. Subsequently, we opened the artificial rainfall device and rained when the SWC decreased to 25.0%Vol ± 2.0%Vol at a depth of 20 cm, which is the multi-year average value of the plow layer in the study area. We scheduled certain rainfall events during the growth periods, including the irrigation recharge and rainfall–runoff experiments. In irrigation recharge, we referred to the daily average rainfall and local irrigation system, and then we irrigated the farmland multiple times to ensure no surface runoff (**Figure 4**). In the rainfall–runoff experiments, we recorded the start time of runoff and monitored the runoff flow rate at intervals of 5 min by the water tank (Lv et al., 2020). The runoff amount is calculated using the following formula:


(3) $$R=\sum_{i=1}^{n}\frac{(r_{i}+r_{i+1})\times T}{2F}\times 10^{-3}$$

Where *R* represents the runoff amount in a rainfall experiment, mm; *n* represents the total number of periods; *r*_i_ represents the runoff flow rate at the time *i*, cm^3^/s; *T* represents the time interval, 300 s; *F* represents the area of the experimental plots, 20.14 m^2^.

#### 2.4.3 Nitrogen and phosphorus detection

We collected the runoff water throughout the rainfall process to quantify the nitrogen and phosphorus concentration at a set time interval (10 or 30 min for short- and long-duration rainfall events, respectively). An alkaline potassium persulfate digestion UV spectrophotometer was used to assess total nitrogen, and the molybdate spectrophotometric method was used to determine total phosphorus (Wu et al., 2021; Wang et al., 2022b). The formula for calculating the total nitrogen and phosphorus loss is as follows:


(4) $$Q=\sum_{i=1}^{n}\frac{\left(r_{i}+r_{i+1})\times (q_{i}+q_{i+1}\right)}{4F}\times T\times 10^{-3}$$

Where *Q* represents the total nitrogen and phosphorus loss in a rainfall event, mg/m^2^; *q_i_* represents the nitrogen and phosphorus concentration at time *i*, mg/L.

#### 2.4.4 Crop growth

We observed the morphological changes of plants by specifications of agrometeorological observation-winter wheat and summer corn (QT/X299-2015 and QT/X361-2016). The observation indicators include plant height, biomass, grain number, grain weight, and yield. The observation method is shown below:

##### 2.4.4.1 Plant height

We randomly chose six summer maize plants from each experimental plot and measured each plant’s height while it was being grouted. Similarly, we randomly selected the four points from each experimental wheat plot and continuously measured the height of 10 wheat plants at each point. As a result, the plant height was the mean value of the observation value.

##### 2.4.4.2 Grain number peer spikes

To determine the average number of grains per spike, when we measured the plant height of winter wheat, we took 100 spikes continuously at each point and a total of 400 spikes. Then, we randomly selected 50 spikes to calculate the average number of grains per spike. In addition, we directly measured the fruit weight on six summer maize plants, so the grain number per spike was calculated from the fruit weight and thousand-grain weight.

##### 2.4.4.3 Thousand-grain weight

We selected 2,000 grains randomly and divided them into two groups. When the weight difference between the two groups was less than 3% of the average, the average value was a thousand-grain weight. If the difference was more than 3%, we took another thousand grains and weighed them, and we used the nearest mean value of the two groups as the thousand-grain weight. The same observation method is used for summer corn.

##### 2.4.4.5 Biomass

The wheat plots were averagely divided into four areas, and each area randomly selected a point. We continuously collected 100 winter wheat plants at every point, for a total of 400 plants, 10% of which were randomly selected for measurement. Subsequently, the samples were divided into stems, leaves, and spikes, placed in the oven, killed green at 105°C for 0.5h, and then baked at 75°C for 6h–12h (Tian et al., 2022). We measured the mass every hour during baking until the weight variation was less than 0.5%. Similarly, we randomly selected six summer maize plants and repeated the above operation.

##### 2.4.4.6 Yield

Referring to the specification, we measured the plant density, grain weight, and thousand-grain weight to calculate the yield (Li et al., 2023). The formula is as follows:


(5) TY=(SGN×TGW×EP)÷1000

Where *TY* represents the theoretical yield, kg/m^2^; *SGN* represents the average number of grains per spike, grain/spike; *TGW* represents the thousand-grain weight, g/1000 grains; and *EP* represents the number of effective plants, plant/m^2^.

#### 2.4.5 Soil microbial sampling

For soil microbial analysis, three soil cores at 10 cm–30 cm depths were randomly collected after the crop was harvested. The soil samples from each experimental plot were mixed completely to form one fresh composite sample that was stored in an icebox. This study focused on bacterial communities because they represented the major microbial communities. The soil samples were sequenced by Majorbio Company through the Illumine sequencing platform (https://www.majorbio.com/). Universal bacterial primers, 338F (primer sequence: ACTCCTACGGGAGGCAGCAG) and 806R (primer sequence: GGACTACHVGGGTWTCTAAT), were used for polymerase chain reaction (PCR) amplification of bacterial communities.

### 2.5 Data analysis

In this study, data were processed by Excel 2019 and plotted by Origin 2020, and ANOVA significance tests were performed by SPSS 25, with statistical significance set at *p*< 0.05 (Zhang et al., 2021a). Finally, we assessed the impact of PFM on the ecological environment of farmland by comparing variations in SWC, runoff, nitrogen and phosphorus loss, the relative abundance of microorganisms, and plant growth. At the same time, we compared the differences in the above aspects between maize and wheat farmland, which aimed to assess the application of PFM in agricultural production and analyze the mechanism.

## 3 Results

### 3.1 Soil water content and runoff

#### 3.1.1 Soil water content

**Figure 6:**
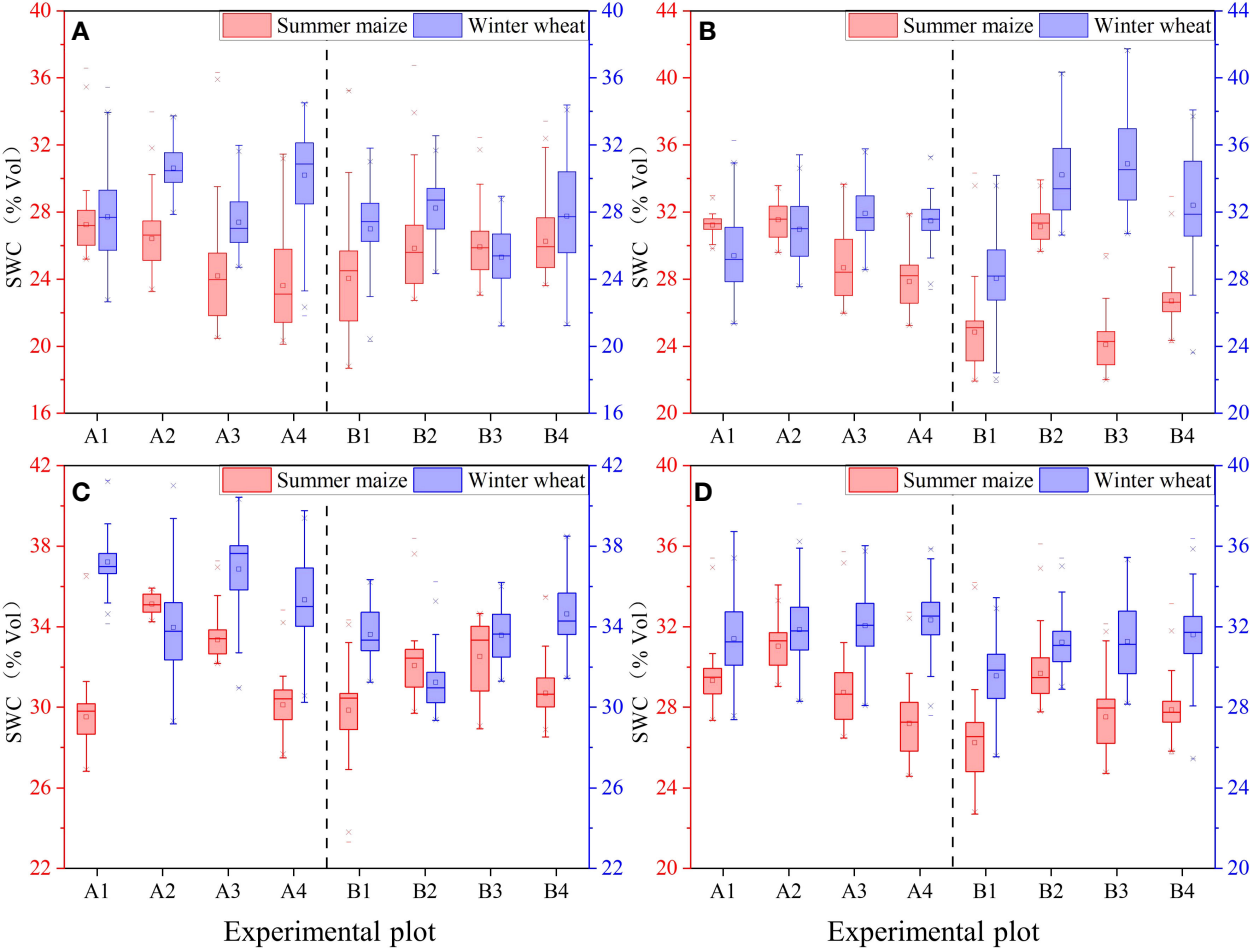
Variations of SWC in different depths (**A–D** represent the SWC in 10 cm–30 cm, 30 cm–50 cm, 50 cm–70 cm, and 10 cm–70 cm depth, respectively).

PFM effectively increased SWC in long-duration rainfall events or on winter wheat plots (**Figure 6**). In detail, PFM embedding increased the SWC of winter wheat at 10 cm–70 cm by 0.7%Vol –2.3%Vol, and the SWC was positively correlated with the PFM volumes (*R* ^2 ^= 0.99 and 0.9). In turn, there were significant differences in SWC between summer maize and winter wheat farmland. In the long-duration rainfall, PFM increased the SWC of summer maize plots at 10 cm–70 cm by 1.3%Vol –3.5%Vol, but A2–A4 groups varied by 1.72.1%Vol, −0.62.1%Vol, and −2.1%Vol compared to the A1 group in short duration rainfall, respectively. The SWC first increased and then decreased with the increase of PFM volume in the summer maize plot.

PFM changed the SWC distribution at different soil depths but had the opposite changing trends between wheat and maize plots. On the summer maize and winter wheat plots, the SWC of the control groups were (25.7%, 28, and 29.7%Vol) and (27.4, 28.6, and 35.3%Vol) within 10 cm–30 cm, 30 cm–50 cm, and 50 cm–70 cm depth, respectively, and the SWC showed a significant difference in the depth of 50 cm–70 cm. Nevertheless, SWC had different changing trends in different soil layers for the long rainfall duration. In contrast, under 100 mm/h rainfall, PFM increased the SWC of summer maize plots by 0.6%Vol–5.6%Vol in 50 cm–70 cm but decreased the SWC by 0.3%–3.5%Vol in 10 cm–50 cm. The variations of SWC in wheat and maize farmland showed the opposite trend when PFM was embedded in the soil, and PFM increased the topsoil SWC but decreased the subsoil SWC. Therefore, some external conditions, such as rainfall duration and crop type, will jointly affect the effectiveness of PFM.

**Figure 7:**
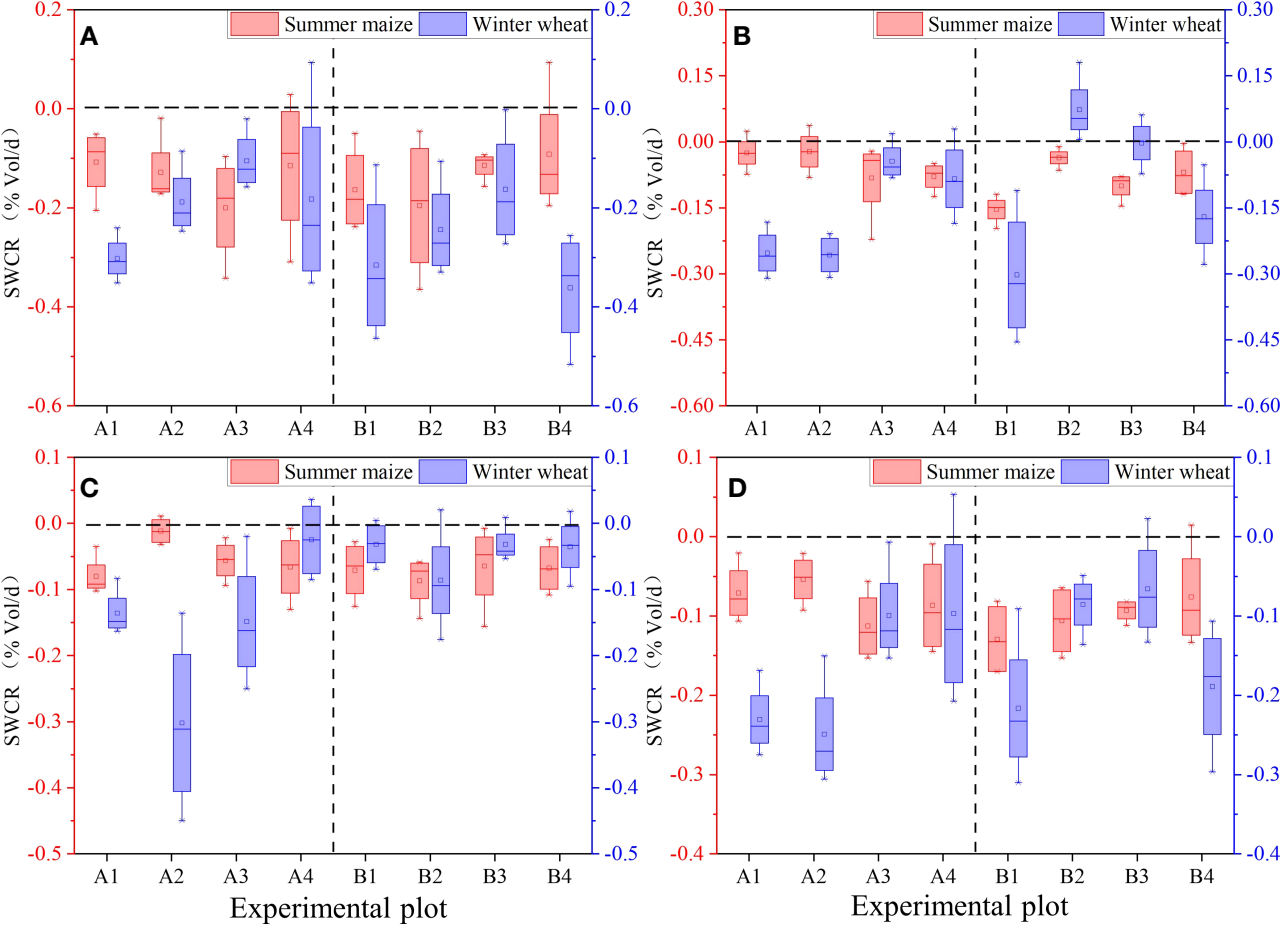
Variations of SWCR in different depths(SWCR indicates the change rate of soil water content per unit time, %Vol/d. **(A–D)** represent the SWCR in 10 cm–30 cm, 30 cm–50 cm, 50 cm–70 cm, and 10 cm–70 cm depth, respectively).

PFM embedding improved soil water-holding capacity (**Figure 7**). In the summer maize and winter wheat farmland, the change rate of soil water content (SWCR) in the control groups was (0.23, 0.13, 0.1%Vol/d) and (0.15, 0.07, 0.07%Vol/d) within 10 cm–30 cm, 30 cm–50 cm, and 50 cm–70 cm depth, respectively. The SWCR was stratified in different soil depths, and the SWCR of winter wheat farmland was significantly higher than summer maize.

Within the 10 cm–70 cm soil depth, PFM decreased the SWCR in winter wheat farmland by −0.02%Vol/d–0.15%Vol/d, compared with the control groups. In contrast, within the summer maize and winter wheat farmlands, the SWCR of both ranged from 0.07%Vol/d to 0.14%Vol/d and 0.07%Vol to 0.25%Vol, respectively, which means that SWC varied more rapidly within the winter wheat farmland. In detail, within the winter wheat and summer maize farmland, the SWCR within the soil depths of 10 cm–30 cm, 30 cm–50 cm, and 50 cm–70 cm was (0.15%Vol/d, 0.07%Vol/d, and 0.07%Vol/d) and (0.23%Vol/d, 0.13%Vol/d, and 0.13%Vol/d), and the SWCR was stratified significantly by 30 cm and showed significant variability between the 10 cm–30cm and 30 cm–70cm soil.

#### 3.1.2 Runoff

**Figure 8:**
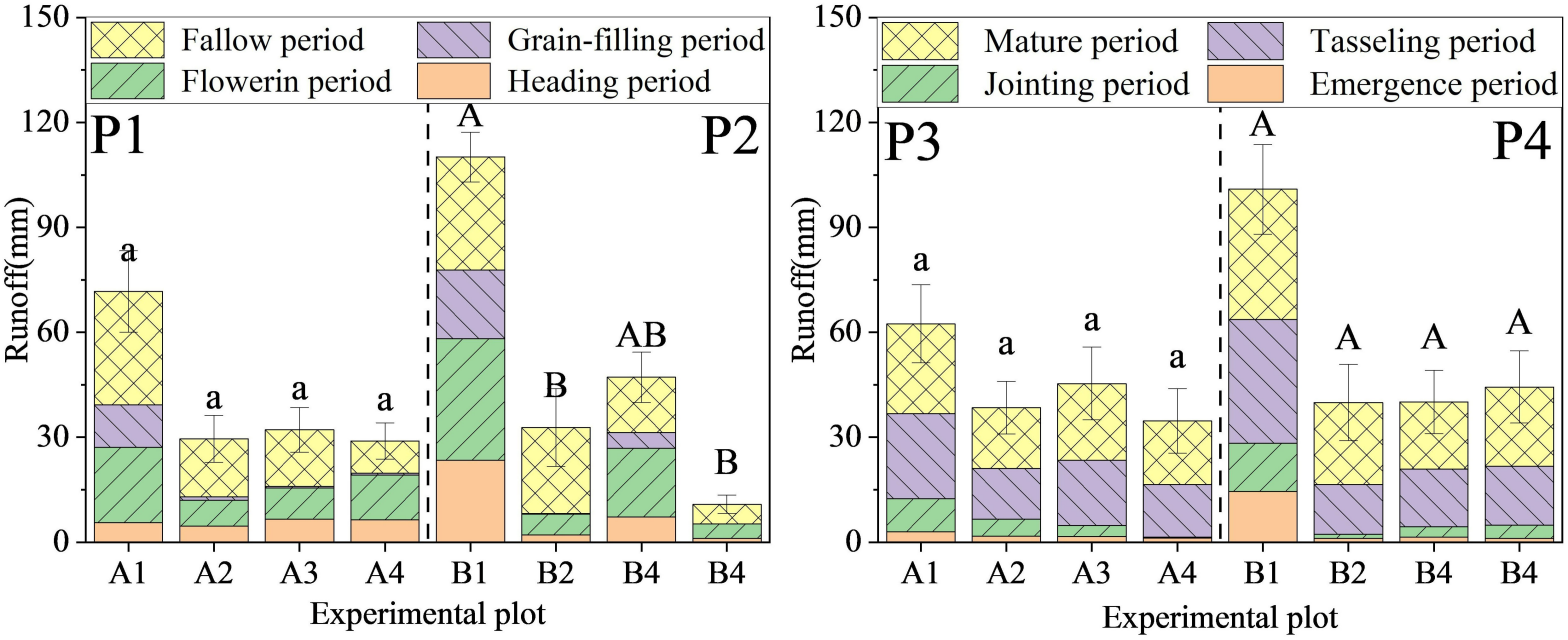
Observed runoffs in the winter wheat and summer maize (P1, P2) in the corner represent 100 mm/h and 50 mm/h rainfall events in the winter wheat experiment, respectively. (P3, P4) represent 100 mm/h and 50 mm/h rainfall events in the summer maize experiment, respectively. (a, b) and (A, B) in the picture indicate the significant difference between the different groups at 0.05 in 100 mm/h and 50 mm/h, respectively).

PFM effectively reduced the runoff. Within the summer maize farmland, PFM embedding decreased the runoff by 27.3%–44.4% and 56.1%–60.4% under 100 mm/h and 50 mm/h rainfall events, respectively, while the reductions in the winter wheat experiment ranged from 49.6%–67.1% and 59.7%–93.3% (**Figure 8**). In other words, the reduction ratio of PFM for runoff in summer maize farmland was lower than that of the winter wheat experiments, and it had a higher reduction ratio for runoff in long-duration rainfall. PFM was more suitable for low-intensity and long-duration rainfall events.

### 3.2 Farmland ecological environment

#### 3.2.1 Nitrogen and phosphorus loss

**Figure 9:**
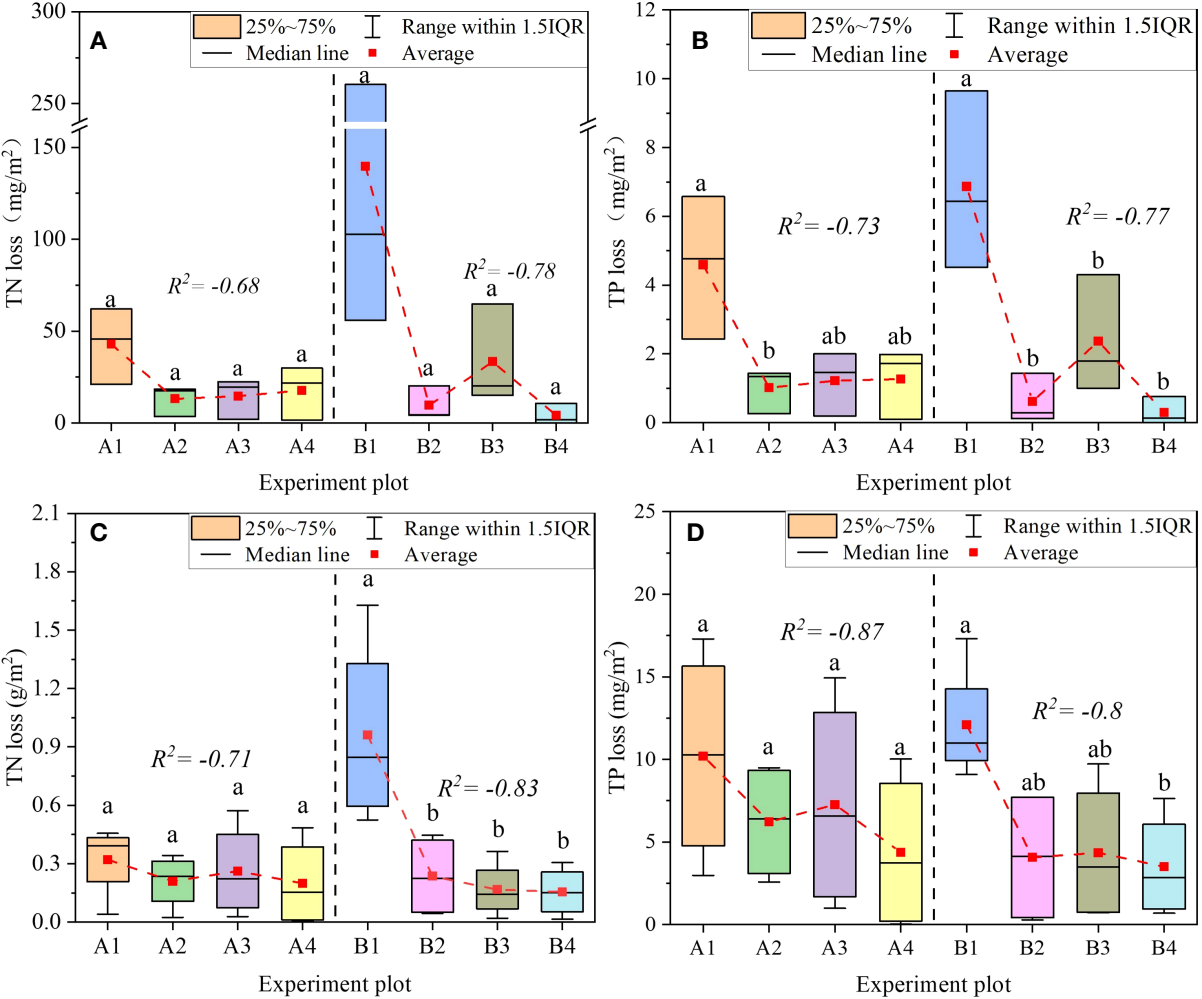
The total loss of TN and TP in the runoff **(A, B)** represents the total loss of TN and TP in the winter wheat experiment, respectively. **(C, D)** represent the summer maize experiment in the same conditions. a and b represent the significant difference between the different groups at 0.05).

PFM embedding reduced total nitrogen and phosphorus (TN and TP) losses in runoff water. In the summer corn experiment, PFM embedding decreased the TN loss by 18.3%–37.9% and 75.5%–83.8% in 100 mm/h and 50 mm/h, and the TP loss decreased by 28.8%–57.1% and 64.1%–71.1%, respectively. PFM had a better ability to retain soil nitrogen and phosphorus elements and had a weaker effect on soil erosion in long-duration extreme rainfall events (**Figure 9**). Compared with the wheat and maize experiments, PFM had a higher impact on TN and TP losses in the winter wheat experiment. In the same volume of PFM embedding, the reduction ratio of TN loss in winter wheat farmland was 13.2%–52.4% higher than that in summer maize farmland (except for the B3 group), but the TP was 1.2%–44.7% higher than in the maize experiments. Therefore, PFM could effectively reduce soil nitrogen and phosphorus losses and have a stronger application effect in winter wheat farmland.

#### 3.2.2 Microorganism

##### 3.2.2.1 Relative abundance

**Figure 10:**
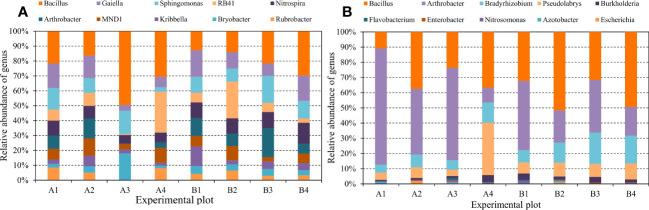
The relative abundance of soil bacterial communities at the phylum level. Pictures **(A, B)** represent the topsoil (10 cm–30 cm) in the wheat and maize experiment.

Plant species, rainfall duration, and PFM volume jointly affected the relative abundance of bacterial communities. In the wheat and maize farmland, the dominant species at the phylum level were Proteobacteria, Acidobacteria, Actinobacteria, and Chloroflexi, which accounted for 9.36%–43.16%, 11.53%–32.57%, 9.43%–30.63%, and 9%–16.14% of the bacterial community, respectively, and the above dominant species cumulatively accounted for 67%–82.18% (**Figure 10**). During the winter wheat experiment, the level of soil bacterial phylum changed to some extent between different treatments. PFM decreased the relative abundance of Proteobacteria and Acidobacteria but increased the relative abundance of Actinobacteria. Meanwhile, the long-duration rainfall events increased the relative abundance of Proteobacteria and Acidobacteria but decreased the relative abundance of Actinobacteria and Chloroflexi. In the summer maize experiment, PFM embedding significantly increased the relative abundance of Proteobacteria but decreased that of Acidobacteria and Chloroflexi, and there was no significant difference in the relative abundance of dominant populations between different rainfall events.

**Figure 11:**
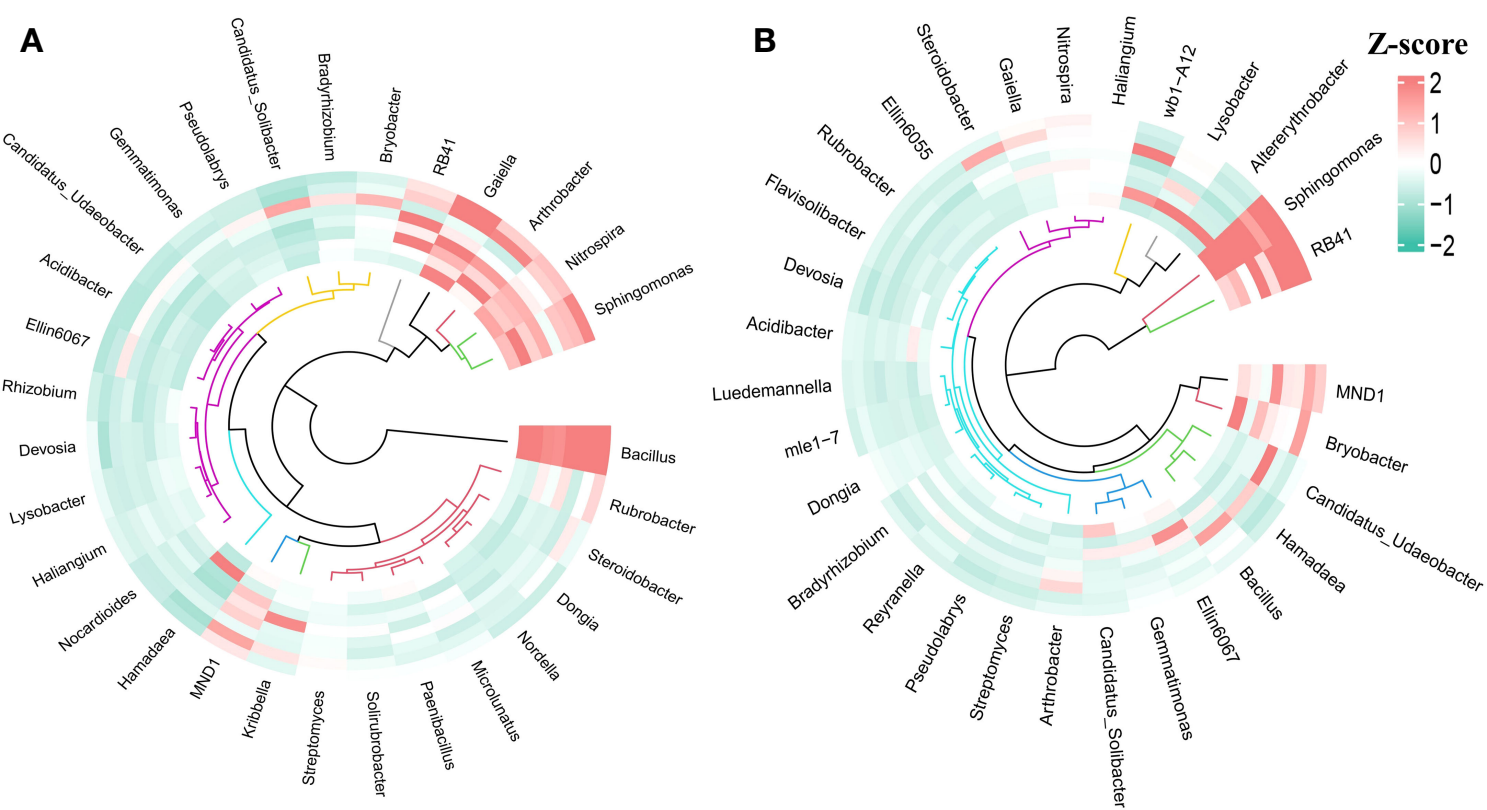
The relative abundance of soil bacterial communities at the genus level. Pictures **(A, B)** represent the topsoil (10 cm–30 cm) in the wheat and maize experiment.

The species composition of microbial bacteria at the genus level significantly differed in winter wheat and summer maize plots. In the winter wheat field, the dominant species at the soil microbial genus level were Bacillus, Gaiella, Sphingomonas, RB41, and Nitrospira (**Figure 11**). PFM embedding increased the abundance of Bacillus and RB41 but decreased the Gaiella, Sphingomonas, and Nitrospira. Conversely, the relative abundance of Bacillus, Arthrobacter, Bradyrhizobium, and Pseudolabrys microbial communities in summer maize farmland was higher. At the same time, PFM embedding resulted in a significant decrease in Arthrobacter strains and different degrees of growth of other dominant strains. Therefore, crop species have significant differences in microbial abundance and species at the genus level.

##### 3.2.2.2 Microbial diversity index

Short-duration rainfall events reduced microbial diversity. The richness of the microbial structures was generally evaluated by several microbial diversity indicators, specifically ACE and Chao. Higher values of these two estimators represent a higher richness of microorganisms. In the summer maize experiments, the microbial diversity index of the PFM groups was lower than the control group (except for the Simpson index, **Table 2**). Conversely, the effect of PFM on microbial diversity showed similar laws in the wheat experiment. The microbial diversity index of the A3 and A4 groups was lower than the A1 groups, but the PFM groups were higher than the control group under long-duration rainfall conditions. In summary, PFM reduced microbial diversity in short-duration rainfall events. The larger the volume of PFM embedding, the greater the adverse effect, but it was the opposite under long-duration rainfall conditions.

**Table 2:** Microbial diversity index.

Experimental Group	Winter wheat experiment				Summer maize experiment			
	ACE	Chao	Shannon	Simpson	ACE	Chao	Shannon	Simpson
A1	3229.85	3245.78	6.43	3.82E-03	4651.44	4555.89	6.76	4.34E-03
A2	3537.58	3535	6.55	3.60E-03	3891.76	3892.28	6.56	3.67E-03
A3	1696.32	1721.89	5.53	8.61E-03	4200.99	4190.24	6.49	5.55E-03
A4	3055	3016.2	6.3	4.47E-03	3731.74	3738.83	6.23	9.07E-03
B1	3481	3446.26	6.43	4.25E-03	4303.56	4340.45	6.73	3.55E-03
B2	3466.42	3434.58	6.59	3.33E-03	3532.52	3538.67	6.05	1.09E-02
B3	3708.81	3663.91	6.43	5.90E-03	4399.17	4353.81	6.51	7.76E-03
B4	3905.78	3830.05	6.58	3.38E-03	3534.29	3521.82	6.02	9.84E-03

### 3.3 Crop growth

#### 3.3.1 Plant height

**Figure 12:**
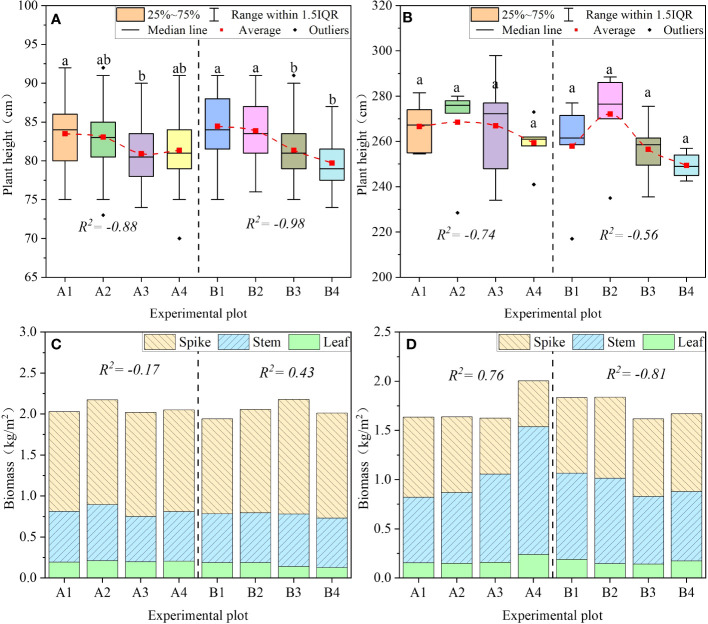
The variation of plant height and biomass. **(A, B)** represent the plant height of wheat and maize, respectively. **(C, D)** represent the plant height of wheat and maize, respectively. (a, b) represent the significant difference between the different groups at 0.05.

PFM embedding decreased the plant height. During the winter wheat experiment, the plant height in the PFM experiment groups decreased by 0.5%–5.6% compared with the control groups, and the volume of PFM was negatively correlated with the plant height of winter wheat (**Figures 12A, B**, *R* ^2^ = −0.88 and −0.98). Nevertheless, there was some significant difference in the summer maize experiment, the plant height showed a trend of increasing first and then decreasing with the growth of PFM volume, and the variation range of the PFM groups was −3.3%–5.5% compared with the control groups (*R* ^2^ = −0.74 and −0.56, *p* > 0.05). Overall, excessive PFM embedding decreased crop plant height under extreme rainfall conditions.

#### 3.3.2 Biomass

Crop biomass was affected jointly by the amount of PFM embedding and the irrigation method. In the mature periods, PFM increased wheat biomass by 0.3%–12.1% compared with the control groups, and wheat biomass increased first and then decreased with the increase of PFM volumes (**Figures 12C, D**). Overall, the increase in wheat biomass was mainly due to the spike weight, which increased by 1.9%–20.7%.

In the summer maize farmland, the stem and leaf biomass in the B2–B4 groups decreased by 4.6%–22.2% compared with the control group. However, the grain biomass increased by 2.4%–20.4%, and total biomass showed a decreasing trend when excessive PFM was embedded in the farmland. In addition, in the short-duration rainfall events, PFM embedding increased the plant biomass in the A2–A4 groups by −0.6%–22.7%, and the main growth comes from stems and leaves. In conclusion, different irrigation methods would affect the distribution of crop biomass, and there was an opposite trend for biomass when PFM was buried in wheat and maize farmland.

#### 3.3.3 Yield

**Figure 13:**
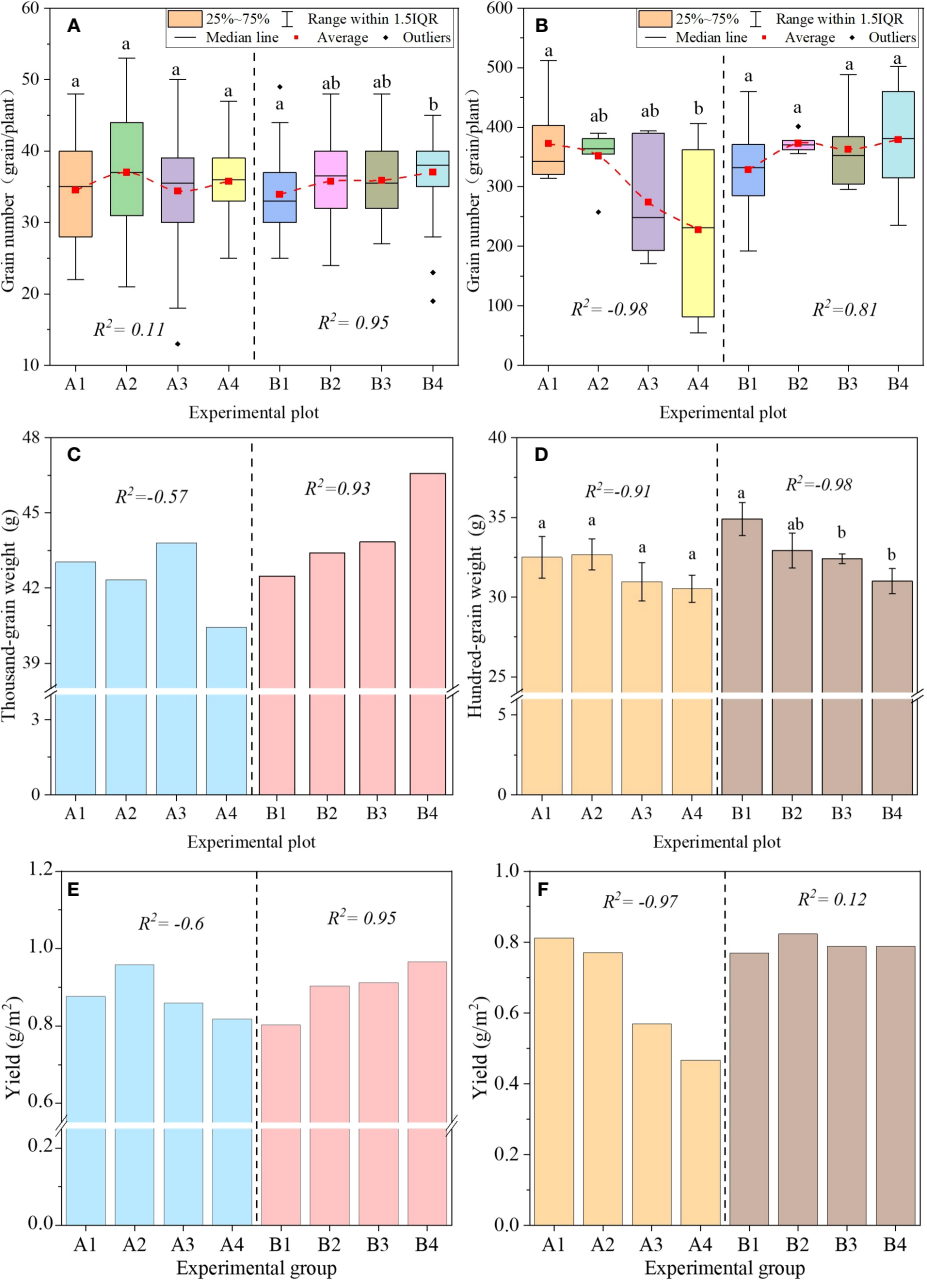
The variation of grain number, grain weight, and yield. Pictures **(A, B)** represent the Grain number of wheat and maize, respectively. Pictures **(C, D)** represent the Grain weight of wheat and maize, respectively. Pictures **(E, F)** represent the yield of wheat and maize, respectively. (a, b) represent the significant difference between the different groups at 0.05.

Long-duration rainfall events increased the grain number but played a negative role under short-duration rainfall events. In the long-duration rainfall events, PFM embedding increased the grain number of wheat and maize by 5.4%–9.2% and 10.4%–15.3%, respectively. The grains number increased with PFM volume (*R* ^2 = ^0.95 and 0.81, **Figures 13A, B**). Alternatively, the effect of PFM on grain number was significantly different in short-duration rainfall events. In the winter wheat experiment, the grain number in the A2 and A4 experimental groups increased by 7.1% and 3.5%, respectively. While the A2–A4 groups decreased by 5.6%–38.8% in the summer maize experiment, the grain number of maize was negatively correlated with PFM volume (*R* ^2^ = −0.98). Therefore, PFM effectively improved the grain number under long-duration rainfall conditions.

Short-duration rainfall events decreased the grain weight when excessive PFM was embedded in the farmland. The result showed that PFM increased the grain weight of winter wheat by 2.2%–9.6% in long-duration irrigation, and the grain weight was positively correlated with the PFM volume (*R* ^2 ^= 0.93, **Figures 13C, D**). But the above pattern was exactly the opposite in other experimental scenarios. PFM decreased the grain weight by 4.7%–11.2% in the summer maize experiment, and the grain weight decreased with PFM volume increasing (*R* ^2^ = −0.91 and −0.98). Meanwhile, in different rainfall events, the grain weight of the B1–B4 groups was higher than the A1–A4 groups by 7.38%, 6.12%, 4.52%, and 1.64%, respectively. Therefore, PFM had a more obvious benefit on grain weight in long-duration rainfall events, but it decreased the crop grouting rate in shorter rainfall events.

There was an interaction between PFM embedding and irrigation methods, which jointly affected crop yield. The result showed that PFM reduced the yield of summer maize by 5.1%–42.5% in short-duration irrigation (**Figures 13E, F**), and there was a negative correlation between the yield and PFM volume (*R* ^2 ^= 0.97). But in the winter wheat experiments, the yield variation in the A2–A4 groups was 9.4%, −1.9%, and −6.7% compared with the A1 group. So, it was easy to make the crop reduction when PFM was heavily embedded in the soil. On the contrary, in long-duration rainfall events, PFM increased the yield of wheat and corn by 12.6%–20.4% and 2.4%–7.1%, respectively. To sum up, we compared the experiment in two crops, and the benefits of PFM embedding in winter wheat farmland were significantly better than those in summer maize farmland.

## 4 Discussion

In this study, the effectiveness of PFM mainly comes from its three characteristics: high porosity, high permeability, and material composition. First, PFM, with high porosity, has excellent hydrophobic drainage capacity. According to the soil moisture characteristics curve, the priority of PFM for water absorption is lower than that of soil, so under the effect of the water potential difference, the water inside the PFM can timely replenish the evaporation of water lost from the PFM surface and the surrounding soil (Lv et al., 2021). Second, high permeability is demonstrated during rainfall, and the excess free water infiltrates the PFM cube. Due to the larger pore structure of PFM, the infiltration rate is higher than that of the soil, which accelerates the vertical infiltration of the soil unit and forms a preferential flow (Lv et al., 2020). Finally, PFM is composed of gold ions, alkali metal oxides, and carbonates, which can adsorb organic carbon from the soil, stabilize agglomerates, and improve soil geotechnical indexes such as enzyme activity, organic matter, humus, and fulvic acid content in agricultural soils (Gu, 2021).

### 4.1 Effect of PFM on the SWC and runoff

PFM could effectively improve the soil holding capacity. In the winter wheat experiment, SWC had a positive correlation with the volume of PFM embedding, but excessive PFM embedding exacerbated the risk of farmland drought in short-duration rainfall events (Ma et al., 2016; Li et al., 2022), which was different from the research results of others. For example, rock wool increased the SWC by 29.2% after a long drought when it was buried in forest land (Gu, 2021). Lv thought the potential energy difference mainly influenced the water exchange between PFM and the surrounding soil. When the SWC is near saturation and the suction pressure is < 75 cm, the free water in the soil will infiltrate into PFM. Afterward, the free water in the soil is absorbed by PFM, and when the water content of PFM reaches 50%, its suction pressure decreases to equal the surrounding soil. Only when the suction pressure of the surrounding soil is < 5 cm can the PFM be fully saturated so that its enhancement of the soil water storage capacity reaches its theoretical value (Lv et al., 2021). However, the above process requires a sufficiently long rainfall duration or large rainfall volume. In other words, under the influence of soil water potential difference, the priority of soil water storage is higher than that of PFM. So, when the embedded volume of PFM is larger, or the rainfall duration is relatively shorter, it is difficult for PFM to fully absorb water, which quickly leads to insufficient water release after rain, or even reduces the maximum saturated water content of PFM, thus increase the risk of drought in farmland (Choi and Shin, 2019; Li et al., 2022; Liu et al., 2022).

The experimental result showed that SWC had a significant difference in deep soil between the different crops because plant root distribution and plant water consumption characteristics made SWC different obviously in the different crops (Hou et al., 2021). In detail, Yang found that wheat derived 41.0% ± 14.9%, 37.2% ± 10.0%, and 21.9% ± 8.9% of its water from the 0 cm–20 cm, 20 cm–50 cm, and 50 cm–160 cm soil depths, respectively (Yang et al., 2018). But the main water uptake for corn root primarily derived water from the 40 cm–60 cm soil layer in whole growth periods (Wu et al., 2016). So, the water uptake characteristics of the root resulted in the SWC having significant differences in 50 cm–70 cm depth. In addition, lime concretion in black soil is also the main factor affecting the SWC. The upper soil has high soil evaporation intensity and poor water retention capacity.

In contrast, deep soil has low soil capillary force and rising height of capillary water, which make it difficult to satisfy the water demand by the plant root in time (Zhang et al., 2001). The above soil characteristics increased the SWC and SWCR with soil depth, and there was a significant difference in SWC and SWCR with 30 cm–50 cm soil depth as the boundary. Alternatively, the SWC and SWCR were low in the summer maize farmland, but the opposite in the winter wheat farmland correlated with the evaporation and groundwater level (Zhao et al., 2020). Specifically, the higher temperature made the evaporation stronger in summer farmland, and the higher groundwater level had a certain supplementary effect on the upper SWC. So SWC and SWCR in summer maize plots were lower than those in winter wheat plots under the joint action of the above factors (Liu et al., 2020a; Zhang et al., 2021c).

PFM embedding effectively reduced the runoff. PFM with enormous pores could enhance the soil porosity, which increased some hydraulic parameters such as field water holding capacity and saturation water content (Lv et al., 2020; Lv et al., 2021). At the same time, PFM with higher permeability lead surface water to infiltrate the soil rapidly during rainfall. As the volume of PFM embedding increases, vertical infiltration of soil units is accelerated and preferential flow is formed, and the reduction of surface runoff will become inevitable with the increase of PFM volume (Li et al., 2022). In addition, vegetation cover is also an important factor affecting the runoff process (Wang et al., 2021). Compared with bare soil experiments, PFM embedding in wheat and maize farmland had a better effect on runoff reduction (Lv et al., 2020). Specifically, higher vegetation cover can improve the soil roughness and weaken the energy of raindrops, which is conducive to reducing erosion formed by raindrop splashing and intercepting runoff, so the vegetation cover has a great influence on rainfall–runoff (Zhou, 2020; Shi et al., 2022; Zore et al., 2022). During rainfall, the differences in vegetation cover and plant height cause the crop canopy to redistribute stem flow and penetrating rain, directly affecting slope runoff formation (Nazari et al., 2019; Yan et al., 2021). But with the obstruction of leaves, the high-density and short-height crops accelerate the dissipation of rainfall energy, which makes it more effective for rainfall interception (Ma et al., 2016). Overall, the vegetation cover contributed to the runoff reduction, which made PFM have a higher runoff reduction percentage on the winter wheat farmland.

### 4.2 Effect of PFM on the farmland ecological environment

PFM effectively reduced the TN and TP loss in the runoff process. Due to the limitation of PFM embedding depth, PFM could not directly decrease the TN and TP concentration in the surface runoff water. In a way, PFM indirectly infiltrated nitrogen and phosphorus from the surface soil layer to the deeper soil layers through leaching (Palla et al., 2010). In addition, PFM delayed the start time of runoff and reduced rainfall runoff, which implied a reduction in the potential for N and P loss with surface production. At the same time, the greater the vegetation coverage, the greater the effect of plants on the interception of rainfall and the reduction of soil erosion (Wu et al., 2020). In this study, the average leaf area index in the summer maize was lower than the winter wheat, and PFM embedding in the farmland of higher vegetation coverage could effectively reduce nitrogen and phosphorus loss, so PFM had a better application effect on the winter wheat (Yan et al., 2021).

In the wheat and maize farmland, the Proteobacteria, Actinobacteria, and Actinobacteria had high relative abundance in topsoil, and they contained many microbial bacteria with potentially fast growth responses to the high SWC. So, the higher moisture fluctuation rate may partially explain the differences found in the original bacterial community compositions (Jurburg et al., 2018; Bi et al., 2019). However, PFM led to variability in the distribution of strains under different crop conditions. For example, it increased the relative abundance of Bacillus, RB41, Sphingomonas, and Bryobacter in the wheat and maize farmland, respectively. The above bacteria have been shown that they can promote plant growth and enhance plant stress tolerance due to the effect of bacteria on nitrogen fixation, cellulose degradation, phosphate solubilization, plant growth hormone, and other aspects (Sonkurt and Cig, 2019; Asaf et al., 2020; Jiao et al., 2022; Sun et al., 2022). Gu also demonstrated that the rock wool was buried in the soil, which increased the enzymatic activity, microbial carbon and nitrogen, and bacterial and fungal abundance of the soil (Gu, 2021). The mechanism of PFM influencing the microorganism’s abundance comes from the following aspects: (1) PFM with high porosity provides a suitable place for microbial reproduction; (2) the improvement of SWC and nutrients is conducive to microbial proliferation; (3) PFM contains mucilage, metal cations, and silicates, were part of the soil complex. And metal oxides can adsorb active organic carbon in the soil, effectively preventing its contact with enzymes and microorganisms, thus stabilizing organic carbon, promoting the formation of soil aggregates, and stimulating soil microbial diversity (Gu, 2021). However, due to the difference in the root, physiological, and biochemical aspects of crops, which made the impacts of PFM on the above bacteria had significant differences. Alternatively, it was also explained earlier that the drought risk was exacerbated when rainfall duration was too short or rainfall volume was low, which greatly affected the growth of microbial communities. Therefore, PFM provides microbial breeding sites, improves soil water and nutrient retention capacity, and promotes the formation of soil aggregates. The above advantages effectively promote the proliferation and reproduction of some microbial strains, which benefit soil nitrogen fixation, phosphate solubilization, and crop growth.

### 4.3 Effect of PFM on the crop growth

In the growth period of crops, PFM effectively improved the water holding capacity and water storage capacity, which was beneficial for the uptake of crop roots and photosynthesis (Gu, 2021; Zhang et al., 2021a). Furthermore, PFM with high porosity could provide the growth channel to the plant root system, which improved the soil environment and reduced plant damage from adversity, ultimately increasing crop biomass (Gu, 2021; Liu et al., 2021). At the same time, PFM embedding positively affected the improvement of soil geotechnical indicators such as enzyme activity, organic matter, humus, and fulvic acid content in agricultural soils. Therefore, improving the plant root environment under PFM embedding benefits crop growth.

In this study, the effect of PFM on biomass showed the opposite trends in summer maize and winter wheat farmland. In long-duration irrigation, enough water and nutrient supply could contribute to the accumulation of dry material in the winter wheat farmland. In summer maize experiments, the irrigation seriously affected the distribution of biomass, which seemed to be related to the soil environment, including SWC deficiency and soil microbial diversity reduction (Qi and Liu, 2020). The structural characteristics of PFM also affected crop yield and biomass under short-duration rainfall conditions. In short-duration irrigation events, a higher amount of PFM embedding is related to lower crop yield. Because the substrate moisture content affects the crop absorption, microorganisms, water stress, and evaporation amount in soilless cultivation, the use of a different irrigation system (watering amounts and frequency) can affect differences in crop growth, fruit quality, and yield (Choi and Shin, 2019). The water absorption capacity of hydrophilic rock wool increases with irrigation time, which makes PFM fully take advantage of water absorption capacity in long-duration rainfall, reducing soil erosion and nutrient loss and finally prompting crop growth (Lv et al., 2021).

Conversely, due to the rainfall duration and the applicability of PFM, it is difficult to absorb the water adequately when massive PFM is buried in the soil, which puts the substrate moisture in an unsaturated state for a long time and decreases the maximum saturated moisture content of PFM. It further aggravates the risk of farmland drought (Choi and Shin, 2019; Li et al., 2023). Specifically, the SWC in 10 cm–30 cm soil decreased with the increase of PFM volume in the summer maize experiments, which influenced the grain filling rate and resulted in grain number, weight, and yield decreases. The SWC deficit might be the main reason for the corn production reduction caused by PFM and short-duration irrigation events. Compared with the summer maize experiments, the single rainfall duration and amount of winter wheat experiments were higher than those of the summer maize experiment, and the PFM is more likely to be effective.

PFM improves the soil environment by increasing SWC and soil pore, facilitating crop root uptake and photosynthesis. Although PFM can promote field drainage and water and nutrient retention, the performance of PFM may not be fully utilized after excessive embedding, exacerbating the risk of drought in agricultural fields and leading to crop yield reduction. Overall, Suitable PFM embedding can effectively improve the soil environment due to water stress, improving microbial diversity and promoting crop root uptake and growth. However, we must recognize that PFM only increases the soil water holding capacity and release the water slowly. In addition, the expense of PFM restricts its application on farmland, and the average price of a PFM ranges from 1,000 to 2,000 CNY/m^3^. Therefore, we can use it in intensive agriculture, economic crop planting, or high-value infrastructure (Lv et al., 2020; Li et al., 2023). To sum up, although PFM has shown excellent performance in some fields, such as soil and water conservation and crop growth, its shortcomings in terms of application and economics also deserve further exploration. So, we should further expand the application fields of PFM, like agricultural production, flower cultivation, and urban greening, which aim to decrease costs and explore the effectiveness and feasibility of PFM in complex environments.

## 5 Conclusion

PFM directly changes soil structure, establishing a hydraulic connection with the surrounding soil, enhancing SWC and nutrient distribution, and ultimately promoting crop production. We conducted artificial rainfall experiments in the field of winter wheat and summer corn. Results indicated that PFM could improve soil water holding capacity while lessening runoff and nutrient loss. Alternatively, the greater the vegetation cover, the greater the impact of plants on soil erosion mitigation. PFM embedding affected soil and water conservation better when vegetation cover was high. The retention of water and nutrients could effectively promote the proliferation and reproduction of some microbes, which had a beneficial effect on nitrogen fixation, phosphate dissolution and plant growth. However, due to its structural properties, PFM could not absorb the water adequately during the short-duration rains, which increased the risk of drought on farmland and reduced the corn yield. And PFM embedding resulted in a maximum increase of 20.4% in winter wheat production under long-term rainfall events. Overall, PFM embedding is beneficial in relieving drought and flooding on agricultural land under a proper irrigation system. This study facilitates the implementation of its extensive application in agricultural production, pollution control, urban landscape, and so forth.

## Data availability statement

The sequencing data generated by this study can be found in the **Supplementary Material**.

## Author contributions

All authors contributed to the study conception and design. Material preparation, data collection and analysis were performed by QT, LS and LW. SA helped to revise the manuscript. The first draft of the manuscript was written by LW and all authors commented on previous versions of the manuscript. All authors read and approved the final manuscript.
